# Dissecting the dsDNA viral packaging machinery: structural evolution and potential for therapeutics

**DOI:** 10.1128/jvi.00951-26

**Published:** 2026-08-24

**Authors:** Makayla N. Leroux, Carolyn M. Teschke

**Affiliations:** 1 Department of Molecular and Cell Biology, University of Connecticut 124501, Storrs, Connecticut, USA; 2 Department of Chemistry, University of Connecticut 193657, Storrs, Connecticut, USA; Indiana University Bloomington, Bloomington, Indiana, USA

**Keywords:** large terminase, molecular motor, DNA packaging

## Abstract

Some double-stranded DNA viruses, such as herpesviruses and bacteriophages, utilize a powerful molecular motor to package DNA into their capsids to liquid-crystalline density. The motor is composed of large and small terminase subunits that bind to a portal complex located at a unique vertex on the icosahedral capsid. The small terminase is critical for recognition of the viral genomic DNA. The enzymatic component of the motor is the large terminase that has two functional domains necessary for ATP hydrolysis and DNA cleavage. Bacteriophage large terminase amino acid sequences are highly divergent, but their overall structural architecture and function are conserved across both tailed bacteriophages and herpesviruses. There are limited antiviral drugs for herpesvirus infections. Therefore, the viral DNA packaging motor is being investigated as an alternative drug target. In this review, we discuss the past and recent genetic, biochemical, and structural research on viral terminase motors that has illuminated their role in DNA packaging and potential as candidates for antiviral drug targeting.

## INTRODUCTION

Prokaryotic, archaeal, and eukaryotic double-stranded DNA (dsDNA) viruses, including giant viruses, share the fundamental objective of packaging their genomic material into a protective proteinaceous capsid for safe delivery to host cells, where viral replication can occur. This review will concentrate on genome packaging in bacterial viruses (bacteriophages or phages), as these have the best-characterized systems, with some information taken from studies of herpesvirus packaging. The process of DNA packaging for the icosahedral-tailed archaeal viruses has not been described by biochemical studies. Rather, the evidence for DNA packaging is primarily inferred from structural and genomic observations (1, 2). Likewise, understanding DNA packaging in the giant viruses remains elusive. Studies suggest that some giant viruses use an FtsK-type motor, while other giant viruses may package their genomes with nucleosomes and co-assemble this complex with the capsid (3, 4). Owing to the complexity of giant viruses, the molecular details are tenuous. Thus, packaging for tailed archaeal viruses and giant viruses will not be described further. For a more comprehensive description of herpesvirus DNA packaging, see the reviews in references 5 and 6.

Bacteriophages (phages), herpesviruses, and the tailed archaeal viruses (2, 7), which are all members of the Duplodnaviria realm, have remarkably similar virion assembly pathways (8, 9). Assembly begins with the formation of a precursor capsid (procapsid [PC]), composed minimally of capsid proteins, scaffolding proteins, and a dodecameric portal protein complex positioned at a unique vertex (10–12). Following PC assembly, the next critical step is the packaging of dsDNA into the PC through the portal vertex by the terminase complex, an ATP-powered molecular motor. The terminase complex in phages consists of two subunits: the small terminase (TerS) that recognizes and binds the viral DNA and the large terminase (TerL) that has nuclease and ATPase activity essential for DNA cleavage and translocation. The herpesvirus packaging motor is tripartite, comprising a possible TerS-like component (in herpes simplex virus-1 [HSV-1] pUL28), a TerL (HSV-1 pUL15), and an additional accessory protein subunit (HSV-1 pUL33) that interacts with TerS and is necessary for DNA cleavage (5, 13, 14). This tripartite complex assembles into the active hexameric terminase ring (15).

## PACKAGING MECHANISMS OF dsDNA VIRUSES

dsDNA viruses that use concatemeric DNA substrates utilize two types of packaging strategies: unit-length and headful. In unit-length packagers such as phages λ (16), P2 (17), and HK97 (18), TerS first recognizes a specific DNA sequence in the viral genome known as the *cos* site. TerL recognizes the TerS bound to the DNA and cleaves it within the *cos* site (18, 19). TerL, now bound to the DNA, then binds to the portal vertex and packages DNA into the capsid until encountering a second *cos* site, upon which it cleaves the DNA, resulting in precisely 100% of the genome being packaged. In phage headful packagers like P22 (20), SPP1 (21), and T4 (22), TerS recognizes and recruits TerL to a specific DNA sequence known as the *pac* site. TerL cleaves the DNA downstream of the *pac* site (23) and then packages DNA until all the available space in the capsid has been occupied, at which point TerL cleaves the concatemeric DNA, resulting in 102%–110% of the genome being encapsidated (24). Following DNA cleavage, TerL remains bound to the DNA and disassociates from the portal, primed to initiate another round of packaging on another PC. For phages that use a *pac* site for terminase binding, the *pac* recognition sequences and the position of DNA cleavage relative to the binding site vary widely. Nevertheless, the packaging strategy is conserved: TerS recognizes a *pac* region and TerL performs a cleavage to initiate packaging, which proceeds until a headful is reached (23, 25, 26).

There are exceptions, however, to these classical *cos* and headful packaging strategies. Herpesviruses are considered headful packagers as they use *pac* sites for recognition and subsequent cleavage of the concatemeric genomic DNA, but like unit-length packagers, they package precisely 100% of the unique coding region of the genome and, additionally, through sensing the pressure of the packaged DNA (27), couple completion of genome packaging to cutting of the DNA at a second *pac* site located within terminal repeat regions at the end of the genome (28–30). Some phages, such as Φ29, synthesize unit-length genomes with a terminal protein attached, rather than concatemeric DNA. These viruses do not require nuclease activity, which in Φ29 results in a TerL that has essentially a vestigial, catalytically inactive nuclease domain that retains the ability to bind DNA (31, 32). The ATPase domain is still homologous to other viral TerL proteins. Finally, there are some phages (T3/T7-like phages and T5) that package genomes containing direct terminal repeats that are precisely cut by terminase to an exact length from concatemeric DNA. Although terminase-mediated cleavage during packaging is clearly involved, the precise mechanisms by which packaging initiation and termination generate these terminally redundant genomes are less understood than *cos* or headful systems (33–35).

Regardless of the strategy employed, these viruses all require both a portal complex embedded at a single unique vertex and terminase proteins for successful DNA packaging. In the absence of either, PCs accumulate and fail to mature into infectious particles (29, 36). The essential role of TerL’s enzymatic activities in infectious virus production, coupled with its structural conservation across dsDNA viruses, has made it a compelling antiviral drug target. Past and ongoing research aimed at elucidating its structure and function continues to deepen our understanding of the process of DNA packaging and inform new therapeutic drug strategies.

## ANATOMY OF THE LARGE TERMINASE

**Fig 1:**
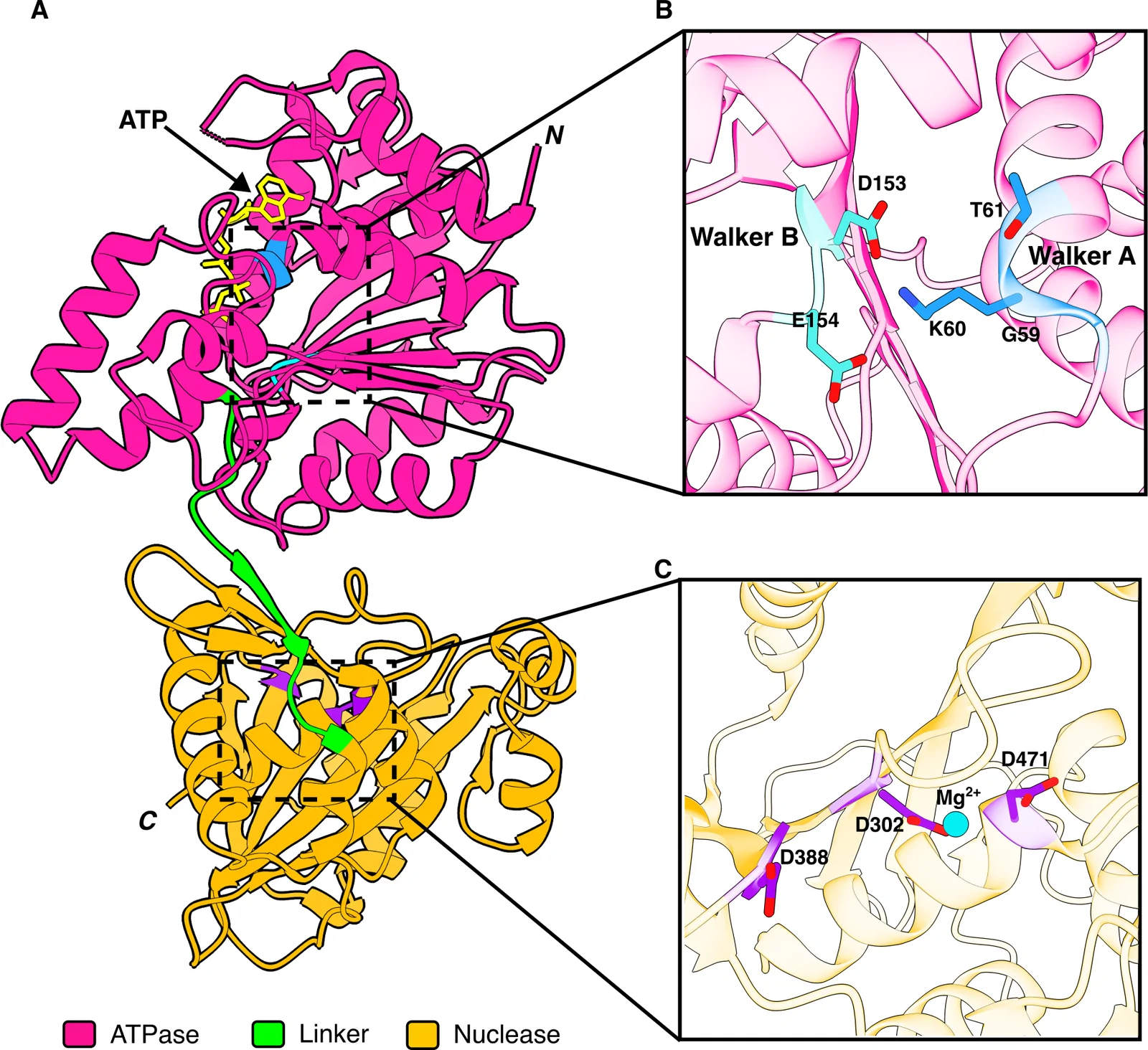
Generalized large terminase architecture. (**A**) The crystal structure of the HK97 large terminase (PDB: 6Z6D). In yellow is ATP that has been modeled using AlphaFold3 (37). (**B**) Important amino acid residues of the Walker A and B motifs in the ATPase domain. (**C**) The acidic amino acid residues comprising the nuclease active site. The crystal structure of the P22 TerL nuclease domain (PDB: 4DKW) was overlaid onto that of HK97 using ChimeraX matchmaker to derive the position of the magnesium ion (blue) in the active site.

TerL has two primary functions: (i) to drive the genome into the head through the portal channel against the increasing pressure of encapsidated DNA via ATP hydrolysis and (ii) to cleave the DNA at the beginning and end of packaging. To perform these functions, TerLs are composed of two distinct domains, an N-terminal ATPase domain and a C-terminal nuclease domain, connected by a flexible linker that is variable in length (Fig. 1A).

### The ATPase domain

TerL belongs to the additional strand conserved glutamate subgroup of ring motors that includes the ATPase associated with diverse cellular activities, a superfamily of proteins that spans bacteria to eukaryotes (38). While proteins of this family have a wide array of functions, they share a structurally conserved ATPase domain (38). For TerL, the ATPase domain is the primary site of DNA binding (39) and contains Walker A and B motifs that are essential for ATP binding and hydrolysis (Fig. 1B). The Walker A motif, also known as the P-loop, of several TerLs follows the classical sequence of -G-XXXX-GK-[T/S]- (X stands for any amino acid) (40, 41), where the threonine/serine hydroxyl coordinates Mg^2+^ of the Mg^2+^-ATP complex, and the lysine ε-amino group coordinates the β- and γ-ATP phosphates to facilitate hydrophilic attack. Deviations from the canonical sequence have also been observed. In phage λ, the sequence is -K-XXXX-GY-S-, where the phosphate-binding lysine is located at the N- rather than C-terminus of the motif. In SPP1, the C-terminal lysine and serine are present, but the glycine that precedes them in the classical sequence is replaced by an alanine (41).

**Fig 2:**
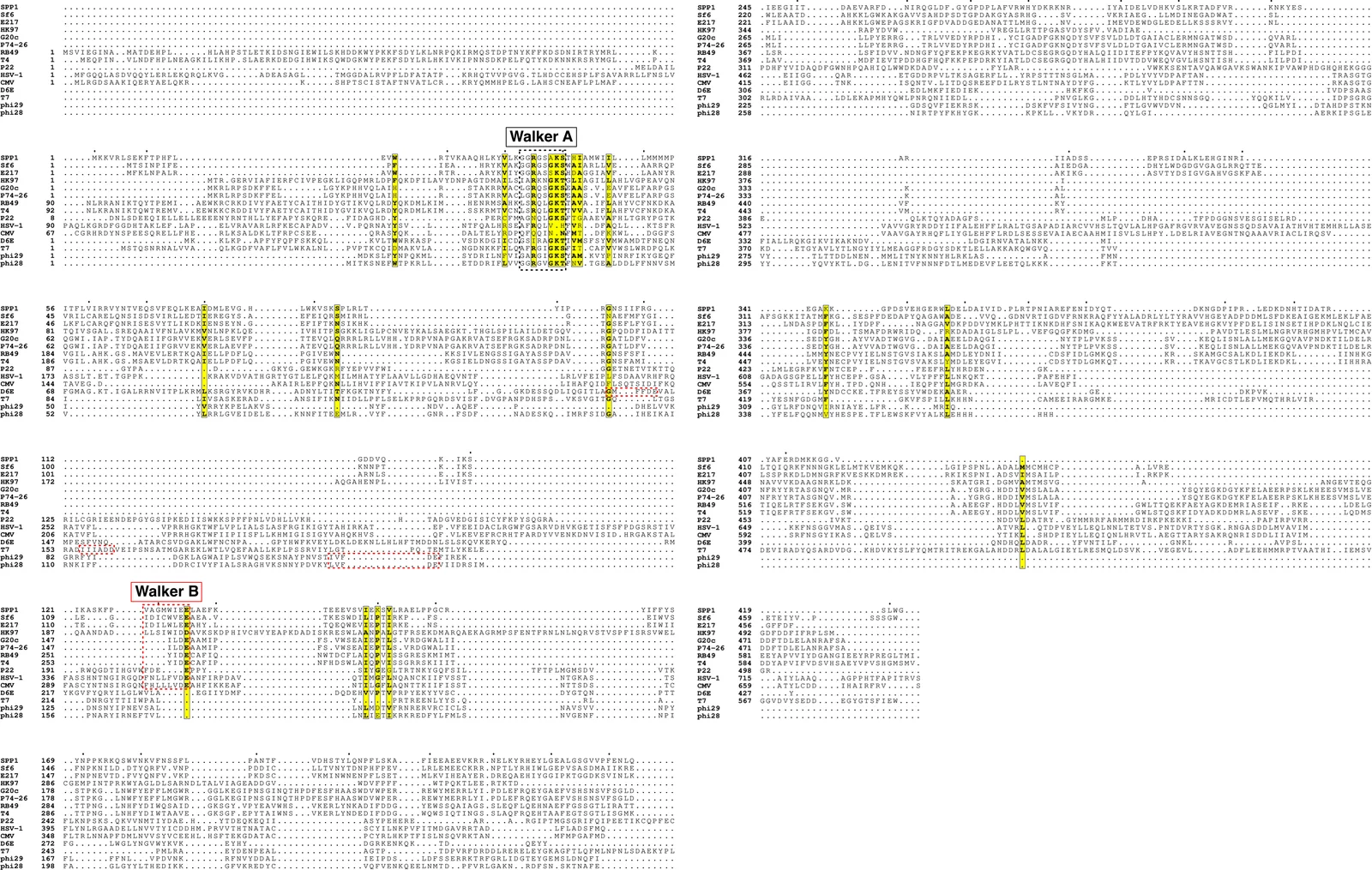
Multiple sequence alignment of the large terminase. The MSA was performed with ClustalOmega using the protein sequences of the large terminases whose structures have been solved in partial or in full. Refer to Table 1 for the complete list. Visualization was performed using ESPript (45). The amino acids in bold, in the yellow boxes, are those that are conserved based on their physicochemical properties. Walker A (-G-XXXX-GK-[T/S]-) motifs are boxed in black, and Walker B (-[R/K]-XXX-G-XXX-LhhhD-) motifs are boxed in red.

Downstream of the Walker A motif in the protein sequence is the Walker B motif (Fig. 2), which has the general sequence of -[R/K]-XXX-G-XXX-LhhhD- (h stands for a hydrophobic amino acid). The conserved aspartic acid residue coordinates Mg^2+^ and activates water for nucleophilic attack. Deviations from this motif have been seen for phage Sf6, where the conserved aspartic acid is a glutamic acid instead (42). Large-scale mutagenesis of these motifs in phage λ revealed their critical roles in ATPase function, demonstrating their importance for ATP binding, motor velocity, and ability of TerL to grip DNA during packaging (40, 42). Mutations to the conserved lysine in the Walker A and glutamic acid in the Walker B motif (Fig. 1B and 2) to alanines abrogate TerL function and are lethal to HK97 phage production (43). Walker A and B mutants in Φ29 have also revealed their importance for DNA binding and ATP hydrolysis, respectively (44).

**TABLE 1:** List of atomic models of the large terminase solved to date

Virus	Part of protein	Structural determination method	PDB code	Reference
HK97	Full length	X-ray crystallography	6Z6D	(43)
Sf6		X-ray crystallography	4IDH	(46)
D6E		X-ray crystallography	5OE8	(47)
T4		Cryo-EM	3CPE	(48)
HSV-1		Cryo-EM	6M5R	(15)
SPP1	Nuclease	X-ray crystallography	2WBN	(49)
G20c		X-ray crystallography	5M1F	(50)
P74-26		X-ray crystallography	5TGE	(39)
E217		X-ray crystallography	8DKR	(51)
P22		X-ray crystallography	4DKW	(52)
RB49		X-ray crystallography	3C6A	(48)
HSV-1		X-ray crystallography	4IOX	(53)
CMV		X-ray crystallography	3N4P	(54)
P74-26	ATPase	X-ray crystallography	4ZNI	(55)
T4	Pentamer	Cryo-EM	3EZK	(48)
T7		Cryo-EM	4BIJ	(56)
Φ29		Cryo-EM	7JQQ	(57)
asccΦ28		X-ray crystallography	7JQ6	(58)

Also critical to the TerL ATPase activity is the motor’s arginine finger. The arginine finger of ringed ATPases is a conserved feature that extends from one subunit across to the neighboring subunit where it inserts into that subunit’s ATP binding site, triggering ATP hydrolysis (59). Once ATP is hydrolyzed, the finger retracts. This orchestrated push-and-pull allows the motor subunits to coordinate the ATP hydrolysis in a sequential motion, converting the energy released directly into the mechanical force required to package the viral DNA into the capsid (57).

### The nuclease domain

**Fig 3:**
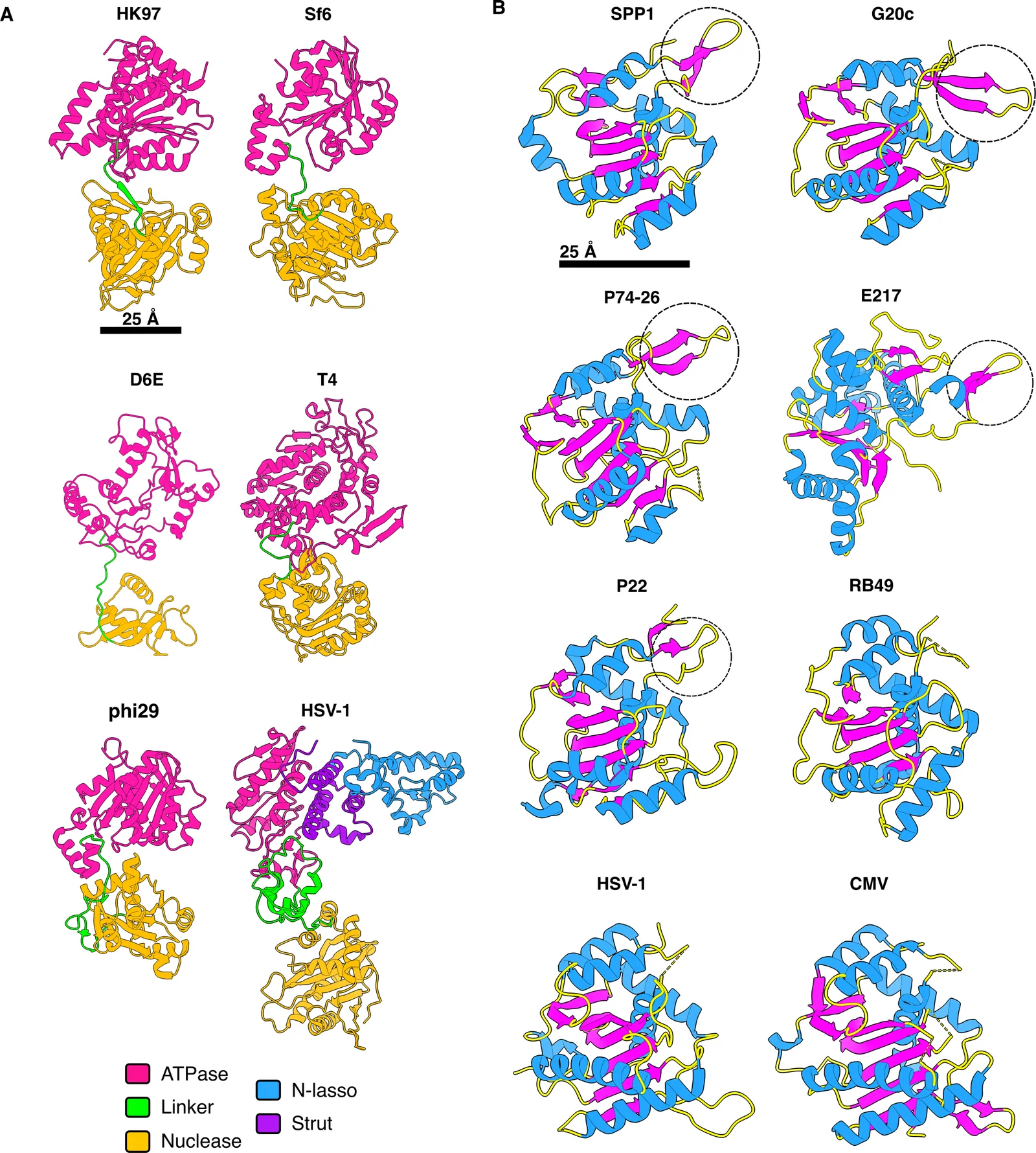
Large terminase protomer structures. Ribbon diagrams of all (**A**) full-length and (**B**) isolated nuclease domain structures solved to date. The HSV1 full-length large terminase in panel A has two additional domains, the N-lasso, colored in blue, and Strut, colored in purple. The protein domains in panel B are colored by secondary structure (helices, blue; strands, magenta; coils, yellow), and the β-hairpin feature is circled. PDB codes for the corresponding models are listed in Table 1.

The nuclease domains of TerL proteins are structurally conserved (Fig. 3) but exhibit limited intrinsic DNA sequence specificity, suggesting that specificity is due to how the nuclease activity is activated rather than from specific binding in the active site. In *cos*-packaging phages, cleavage specificity is imposed primarily through assembly of a sequence-specific binding of TerS to the phage DNA, which positions the TerL nuclease in the correct position for cleavage. In *pac*-site phages, TerS similarly directs the initiation cleavage, but the subsequent DNA cut at packaging termination is triggered by packaging-dependent signals arising from the portal, capsid, and packaged DNA rather than by DNA sequence recognition (23, 25).

The TerL nuclease belongs to the RNase H-like family of endonucleases (48, 54, 60). The RNase H fold is made of a five-stranded mixed β sheet, flanked by α-helices (61). This fold has been repeatedly adapted by alterations that allow it to cleave a variety of nucleic acid substrates, including RNA/DNA hybrids, double-stranded DNA, and structured RNA molecules (60). For example, RuvC, a nuclease that is involved in DNA repair (62), has additional α-helices that surround its central β sheet (63), which are proposed to create a DNA-binding cleft (64). TerL’s nuclease domain structure has evolved similarly, particularly through the addition of a variable-length C-terminal β-hairpin in phage TerLs (Fig. 3B) that is not found in other RNase H-like nucleases. This hairpin structure undergoes conformational changes (49, 65), allowing the nuclease to bind and cleave DNA substrates (49, 50). A structured β-hairpin is not observed in phage RB49 or herpesvirus nuclease structures but rather presents as a disordered loop, suggesting it has a high degree of flexibility (Fig. 3B).

To perform its DNA cleavage function, the TerL nuclease domain uses a two-metal catalysis mechanism that is shared by proteins of the RNase H-like family (50, 52, 66). Conserved acidic residues that coordinate the Mg^2+^ (or Mn^2+^) ions comprise the active site (Fig. 1C). Mutations to these residues abolish the cleavage function of the nuclease (35, 39, 49, 67, 68).

### The two domains work in tandem through movement of the flexible linker

The nuclease domain must cleave DNA at the beginning and end of packaging. How the nuclease activity is regulated to prevent non-specific or premature cleavage is a long-standing question. Biochemical evidence suggests that nuclease function relies on both the presence and activity of the ATPase domain. In phage 74-26, impaired DNA binding by the ATPase domain subsequently inhibits nuclease activity (39), and isolated nucleases from SPP1 and P22 are similarly inactive (49, 52). By contrast, in phage Sf6, the isolated nuclease domain exhibits greater activity than the full-length protein, suggesting that the ATPase and flexible linker confer inhibitory properties against DNA cleavage (46). Despite these opposing observations, both support a model in which the ATPase domain is necessary for maintaining the correct level of nuclease activity.

The linker connecting the two domains plays a key role in signal transduction between them at different steps of DNA translocation. Crystal structures of full-length Sf6 terminase bound to different substrates (ATP, the non-hydrolyzable ATP-γ-S, or ADP) reveal movement of the linker from the ADP- to ATP-bound ATPase domain structures, accompanied by movement of the nuclease domain (46). Similarly, for phage T4, addition of ATP stimulates nuclease activity by causing TerL to adopt a tensed conformation that positions the DNA-binding residues in the nuclease domain for active cleavage (65). Because this tensed state occurs only transiently during rapid translocation, untimely cleavage is prevented (65). Nucleotide-specific conformational changes of phage T7 TerL were also detected by microcantilever assays (69). Here, only ATP elicited an irreversible conformational change on TerL, as observed by a unique bending profile of the cantilever, but the exact changes in the structure were undetermined (69). Combined, these findings suggest that nucleotide-dependent movements within the ATPase domain are transmitted through the linker, repositioning the nuclease domain to facilitate DNA cleavage when necessary. This repositioning, in combination with several potential factors, including internal capsid pressure or portal conformational changes, might allow for the successful second cleavage of the DNA by headful packagers.

## CONSERVATION OF THE LARGE TERMINASE STRUCTURE

Like other viral assembly proteins (70, 71), phage TerLs exhibit minimal sequence conservation (Fig. 2). Conversely, herpesvirus TerLs (e.g., HSV-1 pUL15) are well conserved (72, 73), presumably because of evolutionary pressures on TerL since it binds to many virus protein partners. Phage TerLs also have multiple binding partners; however, their genomes are highly mosaic because of frequent recombination events. Regions of proteins that interact with other proteins often co-evolve, leaving boundaries of mosaicism (74). Regardless, structures of full-length TerLs (15, 43, 46–48) (Fig. 3A; Table 1) and isolated nuclease domains (39, 48–54) (Fig. 3B; Table 1) solved by X-ray crystallography and cryo-electron microscopy (cryo-EM) display a high degree of structural conservation. The nuclease domains of various viruses are practically indistinguishable from one another, with root mean square deviations near 1.0 Å (Table 2), exemplifying an evolutionarily conserved function spanning from the simpler podoviruses to the more complex herpesviruses. Additionally, AlphaFold yields highly accurate predictions for TerL structures when compared to the experimental structural models of HK97 and T4 TerLs, allowing for further study of the evolutionary history of these proteins (75).

**TABLE 2:** Structural similarity of the large terminase nuclease domain determined by root mean square deviation comparison

	SPP1	G20c	P74-26	E217	P22	RB49	HSV1	CMV
SPP1	–^*a*^	–	–	–	–	–	–	–
G20c	1.073	–	–	–	–	–	–	–
P74-26	0.981	0.657	–	–	–	–	–	–
E217	0.993	1.087	1.16	–	–	–	–	–
P22	1.099	1.348	1.219	1.341	–	–	–	–
RB49	1.196	0.894	1.01	1.054	1.234	–	–	–
HSV1	1.42	1.435	1.087	1.046	1.148	0.976	–	–
CMV	1.075	1.024	1.1	1.087	1.209	1.151	0.826	–

^*a*^ – indicates that there is no numerical value for the corresponding cell.

## OLIGOMERIZATION OF THE LARGE TERMINASE

**Fig 4:**
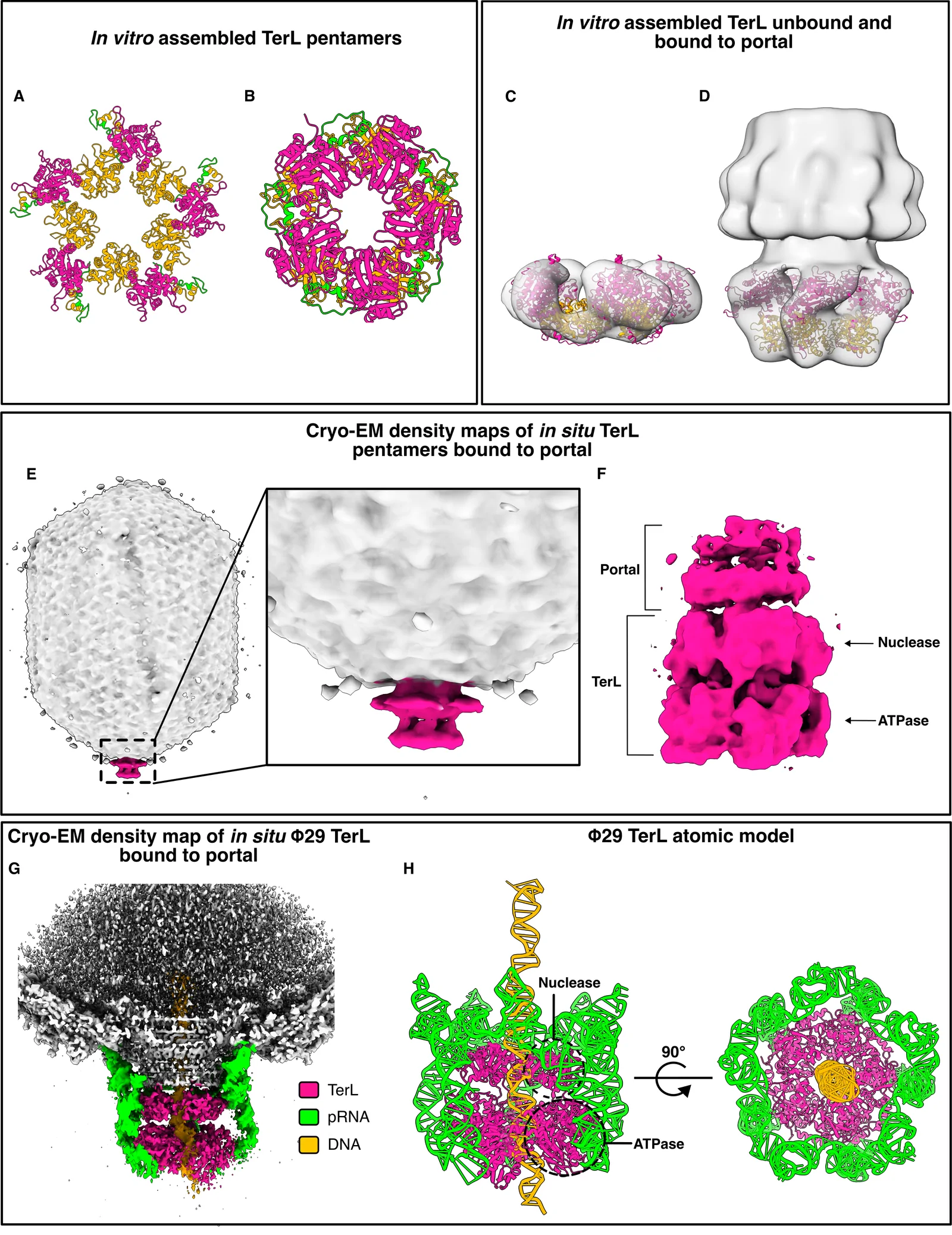
Structure of phage large terminase pentamers. *In vitro* derived structures from phages (**A**) T7 (PDB: 4BIJ) and (**B**) asccΦ28 (PDB: 7JQ6). *In vitro* (**C**) portal unbound (EMDB: 2355) and (**D**) portal bound (EMDB: 2356) T7 TerL complexes. *In situ* TerL capsid-bound structures from phages (**E**) T4 (EMDB: 1572), (**F**) HK97 (EMDB: 16654), and (**G**) Φ29 (EMDB: 22441). (**H**) The atomic model of the Φ29 stalled packaging machinery derived from the cryo-EM density map in panel G. The ATPase domain, nuclease domain, and linker in panels A to D are colored as in Fig. 1.

TerL proteins purified for structural studies have typically been monomeric in solution (15, 43, 46–48). However, the functional state of the motor *in vivo* is oligomeric. *In vitro* analyses of the phages D6E (47), P74-26 (55), asccΦ28 (58), and T7 (56) TerLs by analytical ultracentrifugation, size exclusion chromatography, X-ray crystallography, and cryo-EM, respectively, confirm their assembly into pentamers in the absence of the capsid (Fig. 4A through D). Further *in situ* cryo-EM reconstructions of TerL bound to the capsid in T4 (48), Φ29 (57), and HK97 (76) provide clear evidence for TerL being pentameric (Fig. 4E through H). Divergence from this pentameric oligomer occurs in the herpesviruses, where TerL instead assembles into a hexameric ring of the trimeric protein complex (HSV-1: pUL25, pUL28, and pUL33) (15). This discrepancy suggests a possible functional role for pentamer versus hexamer in phages versus herpesviruses that is not understood.

## INTERACTIONS WITH OTHER MOTOR PROTEINS: A STEP CLOSER TO A BIOLOGICALLY RELEVANT CONFORMATION OF THE LARGE TERMINASE

Due to their structural flexibility, full-length TerLs have been difficult to crystallize without modifications. For many phages, only the nuclease structure has been solved (Fig. 3B; Table 1). The first full-length structure, from phage T4, was only able to be crystallized when the disordered C-terminus was truncated and mutations were made to the ATPase domain, rendering it catalytically inactive (48). Subsequently, the full-length structure of the Sf6 TerL was solved in its wild-type, active form, marking an advance in our structural knowledge of the protein (46). Since then, structures of full-length TerLs have been solved for several viruses (Fig. 3A; Table 1) (15, 43, 47). These structures have provided critical insights into the coordinated roles of the ATPase and nuclease domains during DNA packaging. While TerL has been extensively studied in isolation, it does not act alone to package DNA into the capsid. Therefore, to gain a complete picture of TerL function during DNA packaging, it is pertinent to have structural information of it in complex with its binding partners: small terminase and portal.

### Interactions with the small terminase

TerL interacts with TerS, which forms rings of variable stoichiometry, ranging from a nonamer to a dodecamer, depending on the phage (77–81). Currently, what determines TerS assembly into any particular oligomeric state is not understood (81). The function of TerS is closely tied to the virus’ packaging strategy (11). In *cos* systems, TerS is the master specificity factor that defines both initiation and termination of DNA packaging. In headful systems, TerS primarily functions as an initiation factor, while TerL and the capsid determine when packaging is complete. Because *cos*-packaging systems require precise recognition of specific DNA sequences during both initiation and termination of packaging, TerS proteins in these viruses are subject to strong constraints on DNA-binding specificity. TerS proteins in headful-packaging systems primarily function during packaging initiation at *pac* sites, whereas subsequent cleavage events are determined by capsid filling rather than DNA sequence (11, 82). Herpesviruses have retained the TerL function in a homologous protein (pUL15), but the TerS function appears to have been evolutionarily redistributed among non-homologous proteins, primarily pUL28 and pUL33 in HSV-1 (15, 29).

While TerS is expendable for DNA packaging in *in vitro* phage packaging systems that use pre-cut DNA substrates (83–85), it is essential *in vivo*, where it may modulate TerL activity. *In vitro*, TerS stimulates ATPase activity (78, 86, 87) and impacts nuclease activity (15, 67, 88). Understanding how the conformation of TerL changes in response to TerS to affect its domain activity has been challenging. The two assemble a complex through interactions between the C-terminus of TerS and the N-terminus of TerL (78, 89). The *in vitro* packaging form of this complex in phage λ is a tetramer made of four subunits of one TerL bound to a dimer of TerS (90). However, owing to its transient nature, the structure of the complex has been difficult to establish (91). To date, only low-resolution structures of the TerS:TerL holoenzyme from phage P22 have been solved by cryo-EM (89, 92). The most recent investigation of this complex from P22 suggests that the two proteins can assemble with variable stoichiometry and that the physiologically relevant complex is composed of a TerL pentamer and two TerS nonamers (92). While the pentamer has been confirmed to be the oligomeric state of phage TerLs, it remains unclear how two TerS oligomers would function to deliver DNA to TerL. A high-resolution structure is necessary for further structural interrogation of this complex.

Complexes of TerS and TerL are also observed in herpesviruses, but they form oligomeric assemblies with structures different from that suggested for P22 (15). In HSV-1, rather than an interaction between a TerL pentamer and TerS nonamer, each HSV-1 TerL subunit interacts with a subunit of TerS and a third terminase subunit (pUL33) to assemble a heterotrimer (15). Each of the heterotrimers then assembles into the larger hexameric assembly (15). Alternatively, in Kaposi’s sarcoma-associated herpesvirus (KSHV, a gammaherpesvirus), the TerL/UL15 homolog is ORF29; the TerS/UL28 homolog is ORF7; and the small subunit is ORF67.5, which acts as a structural bridge in the assembly of the motor. ORF67.5 mediates interaction between ORF7 and ORF29. ORF29 also increases the interaction of ORF67.5 and ORF7, suggesting a cooperative binding network is used to build the motor (14). ORF67.5 also contributes to DNA cleavage. Additional studies are required to clarify the active conformational states of the TerS:TerL complex, including the signal that packaging is complete so the final DNA cleavage can occur, or how the terminase motors detach from the filled capsid while maintaining the genome inside the capsid.

### Interactions with the portal complex

Once TerL binds and cleaves the DNA, it must then interact with the portal to pump the DNA into the capsid. *In vitro* genetic and biochemical analyses of complexes from phages T3 (93, 94), λ (95, 96), SPP1 (68, 97), and P22 (98) suggest that TerL interacts with the portal through its C-terminus. When C-terminal residues are deleted, the ability of TerL to bind to the portal is inhibited. The C-terminal region of many phage TerLs is highly charged and contains hydrophobic residues that are likely to mediate the interaction with the portal (98, 99).

Early work on phages T3 (100) and Φ29 (101) indicates that TerL undergoes a conformational change when bound to the portal. These results have since been confirmed structurally with phage T7 proteins (56). Cryo-EM structures of the purified T7 TerL pentamer unbound and bound to portal rings reveal striking conformational differences in the orientation of the ATPase and nuclease domains. When in the unbound state, the domains are oriented next to one another, splayed outwards from the pentamer’s central pore (Fig. 4C and D). In the portal-bound state, the domains rearrange so they are stacked one atop of the other to form a continuous channel with the portal, which is readily observed by an increase in the height of the complex (56). Other than this work on the T7 portal:TerL structure, there is a paucity of atomic resolution structures of the purified complex or an active complex with DNA.

The T7 portal:TerL structure is derived from *in vitro* assembled complexes and therefore may not entirely represent the interaction when the proteins are in the context of the rest of the capsid. To address this, three *in situ* structures of TerL bound to the PC have been solved using cryo-EM. The first of these structures is that of the low-resolution (34 Å) reconstruction of T4 (Fig. 4E) (48). While no specific protein-protein interactions were able to be gleaned because of the low resolution, global conformational switching could be observed. TerL moves from a tensed state, as seen in the crystal structure where the ATPase and nuclease domains are close to one another, to a relaxed state as seen when bound to the portal where the domains are more separated (48). Subsequent single-molecule experiments utilizing optical tweezers served to further refine the mechanism of DNA translocation for T4 (102).

More recently, two additional structures of TerL bound to the PC were solved for Φ29 (Fig. 4G and H) (57) and HK97 (Fig. 4F) (76) at resolutions ~4 and 7 Å, respectively. In the structure of Φ29 during stalled DNA packaging, the nuclease domain assembles a planar ring that interacts with the portal, whereas the ATPase domains are arranged helically, an oligomeric conformation not previously observed in structures lacking DNA (57). The structural insights, in combination with single-molecule force experiments (103) and molecular dynamics simulations (58), led Woodson et al. (57) to propose that DNA translocation is driven by the conformational switching of the ATPase domains from a helical to a planar arrangement, one subunit at a time, until all subunits become planar, allowing TerL to translocate along the DNA, thereby driving it into the capsid (57).

The structure of the stalled packaging motor of HK97 revealed asymmetric binding to the portal, wherein only one of the nuclease domains of the five subunits is in contact with the portal (76). The extended subunit, in contact with the portal, is bound to the non-hydrolyzable ATP. The remaining subunits are bound to ATP or ADP, resulting in more contracted conformations, thereby giving rise to three unique conformations of the TerL occurring at once during translocation. This is reminiscent of the Sf6 TerL, whose domains move in conjunction with the nucleotide substrate that is bound to the ATPase domain (46). The HK97 structure suggests a slightly different model for DNA packaging than that proposed for T4. The HK97 TerL subunits adopt three varied conformations as opposed to a single subunit being tensed and the remaining four being relaxed. Additionally, this structure is incompatible with the Φ29 model of packaging because if each subunit in the HK97 TerL were to transition to the ADP-bound conformation, there would be a complete loss of portal binding. In Φ29, the presence of the pRNAs stabilizing the connection between the portal and TerL allows for binding to the portal even in the ADP-bound state of all TerL subunits. Ultimately, Hawkins et al. (76) propose that the sequential extension and contraction of subunits around the ring provide the movement for translocation (76) for phages like HK97. In total, these structures exemplify the necessity of the conformational switching of TerL between relaxed and tense states to drive DNA translocation. Interactions with the portal, DNA, and nucleotide substrate regulate the different conformational states.

Despite the conserved structures and functions of TerLs, the mechanistic details of how they execute DNA translocation vary between phages. Perhaps this should be expected since each of the phages with solved *in situ* motor structures (48, 57, 76) uses a different DNA packaging strategy: T4 is a headful packager; HK97 uses a *cos* site; and Φ29 uses a pRNA scaffold. Therefore, additional investigation of TerL-bound capsid structures for each type of packager would prove useful for identifying a conserved structural mechanism for translocation among members of that type.

## THE COMPLETION OF DNA PACKAGING

Current TerL-bound capsid structures capture TerL during stalled DNA packaging (48, 57, 76). Structures of TerL in the post-DNA packaging conformation leading to its release have not been published but would be informative. For Φ29, the rate of packaging slows as the TerL translocates the DNA against the building internal pressure of the encapsidated genome (104). For phage P22, increasing pressure is suggested to lead to portal deformation, and portal symmetrization could then lead to the release of the motor (98) and addition of proteins that stabilize the DNA in the capsid. *Cos* site packagers, such as phage λ, work differently. They package DNA until the bound TerL recognizes the second *cos* site and cleaves the packaged genome from the concatemeric DNA. The TerL still senses the increasing pressure in the capsid, but this is not the determining factor for packaging completion; however, evidence suggests that the rate of DNA translocation can affect termination (105, 106).

## THE DNA PACKAGING MACHINERY AS A POTENTIAL DRUG TARGET

Herpesviruses, including HSV, varicella-zoster virus, cytomegalovirus (CMV), Epstein-Barr virus, and KSHV, are widespread and cause a spectrum of diseases that have a significant impact on human health (107). Despite their prevalence, the number of approved commercial drug treatments for herpesvirus infections is limited. One promising target is the DNA packaging motor. For instance, letermovir, an FDA-approved, commercially available small molecule compound, works to prevent the formation of mature cytomegaloviruses by inhibiting DNA cleavage (108). The precise method of terminase complex inhibition is unknown. However, multiple resistance mutations have been mapped to the TerS subunit (pUL56) of CMV, suggesting it is a drug target (108). Recent molecular modeling and docking studies identified the potential binding pocket for letermovir on pUL56 and illuminated its function as a possible allosteric inhibitor (109). The resistance mutations in UL56 lead to deformations in the binding pocket, some of which pushed the drug out of the pocket (110). The downside to letermovir is its limited range, as it affords no antiviral activity against any other herpesviruses, likely due to letermovir’s high specificity in binding (110, 111). For additional discussion on antiherpes drugs targeting DNA packaging, please see the reviews in references 5 and 6.

## CONCLUDING REMARKS

TerL is a core component of the molecular machinery used by both phages and herpesviruses to package DNA into their capsids under extreme pressures. Nearly two decades of structural studies have provided context and corroboration for the early genetic and biochemical work done to investigate TerL. Reviewing this body of work on TerL yields the following conclusions.

1. TerL proteins share a common tertiary structure with some secondary structure variations (e.g., the extended β-hairpin and shortened surface loops in the thermophilic phage G20c nuclease that increase stability at high environmental temperatures) (50).
2. The interdomain communication network between the ATPase and nuclease domains regulates ATP binding/hydrolysis, DNA binding and translocation, and nuclease cleavage activity.
3. Oligomeric states of TerLs differ between prokaryotic and eukaryotic viruses. The reason for this discrepancy requires further investigation.
4. TerL function depends on both transient and stable interactions with TerS, portal, and additional accessory proteins (112) at distinct stages of packaging. Each of these interaction sites could be targets for antiviral drugs.
5. *In situ* structural studies of phages that use different packaging strategies have revealed distinct models for DNA packaging by TerL, indicating mechanistic divergences despite TerL’s conserved structural organization. Further analysis of viruses that use these varied packaging strategies by structural analysis, genetics, microbiology, molecular dynamics simulations, and single-particle biophysics will certainly clarify if there are common or yet uncharacterized mechanisms to be found.
6. The viral DNA packaging complex is a validated antiviral target, exemplified by letermovir’s efficacy against CMV TerS. The structural conservation of TerL makes it an attractive drug target, but further investigation of the druggability of the entire packaging machinery is needed.

Structural characterization to date has provided highly informative but also limited snapshots of full-length TerL from a limited number of viruses. High-resolution structural information of TerL bound to TerS and TerL at the completion of DNA packaging is still necessary to complete the mechanistic picture of the packaging process. Further interrogation of TerL structures will provide insight into the highly conserved process of DNA packaging and assist in the development of novel antiviral compounds.
